# “All is well”: professionals’ documentation of social determinants of health in Swedish Child Health Services health records concerning maltreated children - a mixed method approach

**DOI:** 10.1186/s12887-016-0646-2

**Published:** 2016-08-15

**Authors:** Marie Köhler, Maria Rosvall, Maria Emmelin

**Affiliations:** 1 Department of Clinical Sciences, Lund University, Kunskapscentrum barnhälsovård, Region Skåne, Ängelholmsgatan 1 c, 205 02, Malmö, Sweden; 2 Institute of Medicine, Gothenburg University, Gothenburg, Sweden; 3 Department of Clinical Sciences, Social Medicine and Global Health, Lund University, Malmö, Sweden

**Keywords:** Children, Child Health Services, Health records, Family foster care, Social determinants of health, Child maltreatment, Children’s rights

## Abstract

**Background:**

Knowledge about social determinants of health has influenced global health strategies, including early childhood interventions. Some psychosocial circumstances – such as poverty, parental mental health problems, abuse and partner violence – increase the risk of child maltreatment and neglect. Healthcare professionals’ awareness of psychosocial issues is of special interest, since they both have the possibility and the obligation to identify vulnerable children.

**Methods:**

Child Health Services health records of 100 children in Malmö, Sweden, who had been placed in, or were to be placed in family foster care, were compared with health records of a matched comparison group of 100 children who were not placed in care. A mixed-method approach integrating quantitative and qualitative analysis was applied.

**Results:**

The documentation about the foster care group was more voluminous than for the comparison group. The content was problem-oriented and dominated by severe parental health and social problems, while the child’s own experiences were neglected. The professionals documented interaction with healthcare and social functions, but very few reports to the Social Services were noted. For both groups, notes about social structures were almost absent.

**Conclusions:**

Child Health Service professionals facing vulnerable children document parental health issues and interaction with healthcare, but they fail to document living conditions thereby making social structures invisible in the health records. The child perspective is insufficiently integrated in the documentation and serious child protection needs remain unmet, if professionals avoid reporting to Social Services.

## Background

The links between socio-economic conditions and health have been well documented over recent decades, demonstrating a gradient of health that runs from the top to the bottom of the socio-economic spectrum [1]. Evidence about the social determinants of health has influenced global health strategies [2, 3] and links between social determinants and child health run through many aspects of life, including education, employment, housing, the environment, the economy, school quality and access to recreation and healthcare facilities [4, 5]. Some circumstances within the family [6, 7] have been associated with higher risks of children being maltreated. These include poverty and parental issues, such as psychiatric problems, drug abuse and partner violence as well as the situation in the family’s neighbourhood regarding matters such as alcohol and drug availability, residential instability and unemployment [8]. Maltreatment and neglect during childhood have severe effects on children’s current and future health and well-being [9–11]. A great challenge for healthcare is to prevent and identify maltreatment and neglect during childhood. Through their meetings with children and families healthcare professionals have the chance to identify maltreatment at an early stage [12]. Since it is known that certain social circumstances increase the risk of maltreatment, the professional’s awareness of this is of special interest as it might be the first step in recognizing at-risk children.

Swedish Child Health Services (CHS) have become an important part of the welfare system as the family’s contact with the healthcare system – from the birth of their child until school starts at the age of six or seven years. Although the CHS reach almost all preschool children some vulnerable groups, such as children in foster care, despite their greater health risks, have been found to be less involved in CHS [13]. However little is known about what CHS professionals document about the families’ psychosocial circumstances to obtain information about the setting of the child. We are also unaware if the documentation differs between the most vulnerable children, such as those who are, or have been in family foster care, and others.

*The aim of this study was to investigate what Child Health Service professionals documented in health records about social determinants of health in particularly vulnerable children - children who were to be placed in, or were currently living in family foster care - and to compare with the documentation of children who were not.*

### The Swedish setting

In 1990, Sweden became one of the first countries in the world to ratify the United Nations’ Convention on the Rights of the Child (CRC) [14]. This convention states that every child has the right to “the highest attainable standard of health,” including access to healthcare. One of the articles in this Convention concerns the right to be protected from being hurt and mistreated, physically or mentally (article 19, CRC). In the late 1970s, Sweden was the first country in the world legislating against abuse. Since then, physical punishment of children has decreased [15]. During the same period attitudes have changed and only 8 % of parents support physical punishment, nevertheless around 14 % of Swedish teenagers state that their parents have hit them.

According to UNICEF, Sweden is one of the top five rich countries for promoting the wellbeing of children [16]. As a part of the Swedish welfare system, the CHS provide preventive universal child healthcare and child health promotion to parents and their preschool children, including parental support, regular child health and development check-ups, immunisations and referrals to specialist or inpatient care when necessary. Participation is voluntary and free of charge and CHS reach 99 % of all children in Sweden [17].

When children’s basic needs are not fulfilled by their families, Social Services have a responsibility for child protection provided through a family service system in each municipality. The first step is to support families on a voluntary basis and, if that is not enough, the children can be placed in out-of-home care. It is estimated that around 1 % of children and young people up to the age of 20 in Sweden were placed in out-of-home care during 2012 [18].

Healthcare professionals play an important role in child protection and are obligated to report to Social Services if a child is, or is suspected to be, abused or neglected. There are no systematically and regularly collected national data for the number of reports from healthcare professionals to social authorities. There are even less data regarding the actual number of maltreated children in Sweden. However in 2010, a national survey found there to be considerable differences between regions in reporting frequency of maltreatment. The survey also found that older children were more frequently reported than younger ones [19]. In 52 % of cases, the reasons for the report were connected to parental behaviour, relationship problems, violence, drug abuse, mental health problems, cognitive disparities, sexual abuse and neglect. The survey also revealed that the healthcare sector was less likely to report suspected abuse or neglect than other sectors, such as the police or schools. Swedish research confirms that reports from the healthcare system greatly underestimate the prevalence of maltreatment and neglect [20, 21]. In general, reporting levels are also low among healthcare professionals in other countries [22]. This low reporting rate might be due to several factors, including low awareness of the signs of child maltreatment and unfamiliarity with the reporting processes. There may also be doubts about the benefits of reporting, deficient professional support and lack of knowledge about the conditions and mechanisms influencing child maltreatment.

## Methods

### Overall study design

We chose to retrospectively analyse documentation in CHS health records by health professionals using a comparative approach. This gave an opportunity to compare documentation about children who had been placed in foster care due to maltreatment or neglect with a group who did not have this experience. A mixed method approach was applied that included qualitative content analysis as described by Graneheim & Lundman (2004) complemented by descriptive quantitative information about selected concepts documented in the health records [23]. The benefit of this approach was that it encompassed different perspectives and enabled us to create a more complete understanding of the complexity of the phenomena explored by this study [24].

### Sampling and data sources

The inclusion criteria were all Malmö children 0–16 years of age, who on 15 September 2008, had been placed in family foster care by the Social Services for a period of at least 3 months, but whose parents were still their legal guardians. At this time 223 children met the criteria. For research purposes, informed consent to access the child’s health records was provided by their parents. When the child was aged 15 or above, they could provide their own consent.

**Fig. 1:**
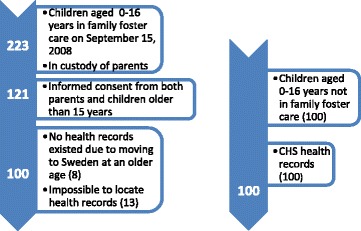
Number of sampled children and data sources

In total, consent was given for 121 of these children. However, 13 CHS records could not be located neither within the healthcare nor in the regional archive and further eight records were lacking since they concerned children who arrived to Sweden *after* the preschool period. Thus, the case group material consisted of 100 CHS health records for children born between 1992 and 2008. CHS health records for the comparison group, who had not been in foster care during the preschool period or when the study started were selected by matching the cases for age, gender and municipality (=Malmö). These records were collected in de-identified format from the regional archive where health records are to be sent when the child has started school. The comparison records were randomly picked by including the first match found (Fig. 1). The CHS documentation for both groups concerned the preschool ages. For the case group the documentation referred to periods *before, during and/or after* family foster care placement.

In total, documentation for 100 children in the case group – 55 boys and 45 girls – was accessed and as many in the comparison group. Fifteen of the 100 records for the children in the case group were incomplete, due to missing documentation. The 100 health records for the comparison group were complete. These data have also been used for a case-referent analysis focusing on the health of children in family foster care [13].

### Health records

Swedish health records are part of its national system. The records were in paper format and should have followed the child from health centre to health centre. On the child’s first visit to CHS, the nurse registers certain psychosocial data, such as family demographics. In addition, child health indicators such as breastfeeding, parental smoking and child development and healthcare issues such as treatments, contacts and referrals should be noted at visits by the nurse or physician as well as free text observations. The health record usually contains documents from the maternal health visits and obstetrical care, as well as from paediatric clinics and referrals and therefore the notes also are made by specialists such as physicians, psychologists and speech pathologists.

### Data analysis

**Fig. 2:**
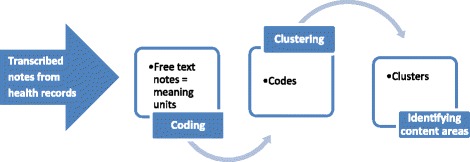
An overview of the first steps in the qualitative content analysis process

The analysis began by reading the total content of all the CHS health records, including the standard sections, free text notes and additional documents. The focus of this study was to understand how the professionals wrote about different types of social determinants of health concerning the child. This means that documentation relevant to different levels – individual, community or societal – of the social determinants of health was included. Notes about the child’s or the parents’ psychosocial health, the family’s social network eg, indicating the involvement of parents, siblings or relatives as well as psychosocial life style matters and societal and environmental issues, were included. Other data influencing the psychosocial environment of the child and its family such as the child’s or its family’s contacts with other healthcare, were also collected. Another part of the data was the professionals’ documentation concerning their contacts by phone or in writing with the families as well as any contacts, including personal meetings, with Social Services or other healthcare functions. The text about these psychosocial matters was transcribed and entered into Open Code 4 software to facilitate the analysis process [25]. The data about children in the case group and the comparison group were entered into separate files. All free text notes were subjected to a qualitative content analysis described by Graneheim & Lundman [23]. The qualitative content analysis was conducted for both groups but focused specifically on the content of the health records of the children in family foster care group. There was no need to condense the meaning units because they were all short and concise. Figure 2 provides an overview of the beginning of the analytical process where the notes of both groups were coded and clustered into content areas relating to our research questions. In Table 1 we illustrate the relationship between one of the identified content areas, the sub-content areas and the associated codes. The analysis continued with a more inductive approach where the data from the case group was interpreted for its manifest meaning by developing categories and sub-categories in relation to the identified content areas [23]. The coding of the health records of the children in the comparison group was integrated into the analytical process to mirror and compare differences, similarities and neglected areas in the documents of the two groups. The pre-understanding of CHS work of one of the authors (MK) having many years of experience as a CHS paediatrician was taken into consideration. This was complemented by the other authors’ long experience of both quantitative and qualitative research. The analytical process was continuously reflected upon and revised by the research group to ensure consistent interpretations. The preliminary coding was mainly performed by the first author (MK). The codes were then read by the last author (ME) and clustered in content areas agreed upon. Counting of codes in the content areas was performed by the first author (MK) while the interpretation and synthesis again was a joint venture including all authors (MK, MR, ME).

**Table 1:** An illustration of the links between codes and the content area THE CHILD based on all health records in the case group

Code	Sub-content area	Content area
Good girl.	Behaviour	THE CHILD
Biting and fighting at preschool.		
Plays with other children.		
Concentration problems.	Psychological issues	
Fantasises about father.		
Attaches to mother.		
Inpatient care at children’s hospital.	Medical issues	
Father has noticed the child squinting.		
The mother has hepatitis C but the child does not.		
Speech is OK.	Development	
Delayed psychological development.		
Positive development in foster family.		
Breastfeeding is ok.	Nutrition	
No appetite.		
Eats the same food as family.		
Enjoys preschool.	Preschool	
Functions well in groups of children.		
No preschool yet.		
Plays outdoors every day.	Leisure	
Takes dance lessons.		
Father says the son likes cars.		

The next phase for both groups included a simple word count of the documentation as well as a description of the distribution of the codes divided into the four content areas identified in the qualitative analysis. In this analysis the 15 incomplete health records in the case group were excluded as well as the corresponding 15 matched health records of the comparison group.

Finally in the discussion, the findings were integrated using the metaphor of triangulation to compare the quantitative and qualitative analysis of the same data, in order to develop a deeper theoretical understanding of the CHS documentation in terms of both content and space [24].

### Ethical considerations

This study aimed to generate knowledge about the documentation in CHS health records, specifically focusing upon vulnerable children who had been placed in family foster care at some point during childhood. Special steps were taken to maintain the confidentiality of personal information about children and parents and to protect the integrity of the professionals involved. No individually identifiable data are therefore published. In addition, informed consent was collected from the parents and any child over the age of 15 years in the case group. However, informed consent was not necessary for the comparison group records as they were supplied in a de-identified format by the regional archive. Ethical approval was received from the Ethic Board in Gothenburg on 15 December 2008 (Ref.no.: 633-08) with additional approval on 26 November 2013 (Ref.no.: T946-13).

## Results

As already shown in our previous study, 70 % of the children in the case group were placed in out-of-home care for the first time during the preschool period (up to 7 years of age) [22]. The median age for the first out-of-home placement was three-and-a-half years and the most common reasons were parental alcohol or drug abuse (35 %), neglect (30 %), parental mental health problems (13 %), parental illness (7 %) and/or child physical abuse (6 %). At least one parent had died for 25 % of these children.

The CHS documentation originated mainly from CHS visits and was primarily made by nurses and fewer by physicians, apart from the documents from inpatient care or clinical health records. These were instead mostly made by physicians or by psychologists, dentists or speech pathologists. The total number of words covering data on social determinants of health at different levels in the free text notes of the 85 complete health records in each group was 2.6 times higher in the case group than in the comparison group (20,663 versus 7987 words).

The presentation of the results will henceforth be structured under the four identified content areas: the child; the family; living conditions and actions by professionals and will integrate the findings from the quantitative and qualitative analysis. The frequencies of codes relating to the identified content areas are shown in Table 2, while Table 3 shows the relationship between the content areas and the categories and sub-categories depicting the manifest meaning of the text and developed mainly for the case group. The presentation will mirror these findings with the results from the comparison group.

**Table 2:** Number (percentage) of *codes* in the four content areas of 85 complete health records from each group

Content areas	Children in family foster care group	Children in comparison group
	*N* = 4080 n (%)	*N* = 1881 n (%)
THE CHILD	849 (21 %)	486 (26 %)
THE FAMILY	1359 (33 %)	794 (42 %)
LIVING CONDITIONS	256 (6 %)	51 (3 %)
ACTIONS BY PROFESSIONALS	1616 (40 %)	550 (29 %)

**Table 3:** Overview of codes, sub-categories and categories concerning the four content areas identified in the documentation of the children in the family foster care group

Content area	Code	Sub-category	Category
THE CHILD	*Stubborn boy.*	*The problematic child.*	Lack of child perspective.
	*Feels bad.*	*Value-oriented assessments.*	
	*Misses dad who moved.*	*Few references to the child’s personal arena.*	
	*Aggressive to little brother.*		
	*Mother thinks MM is annoying at home.*		
	*Defiant girl acting out at home.*		
THE FAMILY	*Parents separated.*	*The chaotic family.*	Parents’ problems dominate.
	*Got stepfather.*	*Parental health problems.*	
	*Mother disappeared.*	*Rocky road of daily life.*	
	*Lives with grandparents.*	*Parental worries.*	
	*Mother physically abused.*	*Absent fathers.*	
	*Mother mental problems.*	*Lack of social support.*	
	*Both parents cognitively disabled.*		
	*Father detoxified.*		
	*Mother obsessed with child’s eye problems.*		
	*Mother 20 years of amphetamine use.*		
LIVING CONDITIONS	*Complex social situation.*	*Misery.*	Invisible social structures.
	*Social problems.*	*Frequent moving.*	
	*Mother tired worried about finances.*	*Unstable living accommodation.*	
	*Parents unemployed.*	*Lack of socio-economic information.*	
	*Mother and child without housing.*		
	*Father and son now living under hard and uncertain conditions.*		
	*Living at different friends’ places.*		
	*Housing problems.*		
	*Family moved?*		
ACTIONS BY PROFESSIONALS	*No answer when phoning.*	*Communication problems.*	Limited focus on child protection.
	*Letter to mother to contact.*	*Focus on medical problems.*	
	*Gave advice to train language and to set limits.*	*Counselling “top down”.*	
	*Preschool professionals are worried.*	*Professional worries.*	
	*A neighbour thinks that MM is being maltreated.*	*Neglecting reporting to Social Services.*	
	*Everything is OK.*	*“All is well”.*	
	*All is well.*		

### The Child

The codes in this content area consisted of 21 % of the total number of codes in the documentation of the case group.

The identified sub-categories showed a problem-oriented approach where parents were concerned about health or developmental issues regarding the child (*“mother thinks MM is annoying at home”, “aggressive to little brother”*) and professionals tended to comment in a value-orientated way (*“good girl”, “stubborn boy”*). The child was described as having problems or even *being* the problem, when it came to development or behaviour. There were few references to the child’s personal life in that the child’s experiences of family life were not noted and documentation on the child’s own arena – preschool – was scarce. In the specific space in the health record concerning preschool the name of the preschool or child care the child attended was noted for 69 % of the children in the case group. Leisure activities were often omitted. The category Lack of child perspective illustrated the insufficient description of the child’s own arena as well as the rare note of the voice of the child. This indicates that the child is not regarded as an active subject and not listened to. The voices in the documentation were instead the adults’ – parents’ or professionals’.

### The Family

With 33 % this was the second largest content area regarding number of codes in the case group. The identified sub-categories showed very unstable and chaotic family situations with frequent conflicts, violence and separations as well as new family members. Legal issues concerned custodial conflicts and, in some cases, investigation of paternity. Fathers were absent both in visiting the CHS and in daily life. Social support consisted of relatives coming along to CHS visits or providing practical support, such as looking after the child or offering housing. Some support, such as domestic help or family education, was delivered by Social Services. The notes showed a *lack* of social support in spite of great and serious needs like homelessness and financial problems. The category Parents' problems dominate summarised overwhelming and severe parental health and social problems, including physical and mental health and cognitive problems together with smoking habits, alcohol and/or drug abuse. Parental worries concerning health or social problems consisted one sub-category. In summary also this theme illustrates that the child had limited room in its own health record. As a nurse commented regarding the medical test PKU, which is supposed to be performed on every newborn child: *“Turns out that not even PKU test has been performed. Probably forgotten due to everything that has been in focus and has taken time concerning mother.”*

### Living conditions

As already reported in a previous study [13], the family demographic data that should be noted in a specific area in the health record concerning parents’ occupation were missing for 72 % of the mothers and 75 % of the fathers in the case group. In the free text this content area was the one least paid attention to (6 % of the codes) in the case group documentation. The notes were generally problem focused and most of them concerned frequent moving eg *“recently moved here”*, *“family moved?”*, *“moved with brother to mother”* and *“moved to a foster family”*. In spite of few notes a picture of a hard life with social misery for these children was revealed both by general or specific notes, eg, *“complex social situation”*, *“living at different friends’ places”*, *“mother and child without housing”*, *“father and son now living under hard and uncertain conditions”*, *“living at a protected housing”, “mother tired worried about finances”* and *“parents unemployed”*. Residence permit was an unusual note. The category Invisible social structures was supported by four sub-categories of which one indicated a prominent lack of socio-economic information about the whole family situation. Altogether, general and specific comments painted a picture of a difficult life full of social misery for the children and their families marked by unemployment and economic problems.

### Actions by professionals

The fourth content area embraced 40 % of the codes in the case group documentation, thereby constituting the largest part. The communication between the family and the CHS professionals during and between the visits dominated, eg *“conversation with father”*, *“gets new time for appointment”* and *“mother called wants home visit”*. A great part revealed communication difficulties and documented attempts to contact the family, eg, *“no answer when phoning”* or *“letter to mother to contact”*. Some notes were made about communication problems due to deficiencies in language comprehension in parents. No interpreter was used in those cases. Counselling during visits was noted concerning eg, parenting, sleep habits, eating, behaviour of the child and medical issues. One nurse noted: *“we talk about activities* eg*, reading to him or kicking the ball”*. Sometimes it was clear that the counselling was initiated by the professional and a “top down” approach could be identified. Another nurse noted *“gave advice to train language and to set limits”* regarding a child of two and a half year who was described as *“aggressive”*. In other cases a supportive approach was obvious, eg, when a nurse recommended the mother to visit the open preschool to establish contact with other families. Contacts and interaction with other healthcare, social functions or Social Services were also noted and concerned telephone calls, meetings with and visits in other healthcare or social institutions eg Child Psychiatry Services, preschool and Social Services. Notes on professionals’ worries were generally rare and were almost exclusively made by nurses and described worries both directly: *“I am worried that MM doesn’t get stimulation”* and indirectly: *“Long conversation about babies’ development and skills”.* A few comments revealed other professionals or persons being worried: *“Preschool professionals are worried”* and *“A neighbour thinks that MM is being maltreated”*. The notes could also indicate that nurses have actively taken a stand for the child. For example a nurse noted several times that the child seemed frightened and she then confronted the father with this fact telling him that the child reacted as if he had been hit. No report to the Social Services was documented being made in connection to this. Another example was a nurse’s notes regarding a little girl: *“I am worried about the girl not getting the stimulation and care that she needs. I shall ask the Social Services for advice. I will make a report hoping that the girl and the family might get the support needed.”* Professionals reporting to the Social Services were rare findings and only occurred for nine of the children in the case group.

The category Limited focus on child protection indicated that professionals did not fully act on information about the child’s severe psychosocial situation and living conditions or signs of maltreatment or neglect. This category was supported by sub-categories showing eg, communication difficulties with the families causing unstable contacts, as well as professional worries but still focusing on medical problems and neglecting reporting to the Social Services. Generally professional worries seemed to be suppressed and/or instead expressed through counselling or interaction with other healthcare or social functions, but not leading to reports. Furthermore, a frequent comment in the free text area was *“all is well*” and *“everything is ok”* and similar formulations. This kind of notes were often seen as the only documentation from a health visit, except for the child’s weight and length, and constituted the greater part of notes made by the physician. This specific notation was used 146 times in documentation of the case group. Table 4 shows notes made by a nurse in a health record in connection to six consecutive visits with a couple of weeks in between. The child was living with its biological family and “social problems” had previously been documented. To note “all is well” after a visit shortly before receiving information that the mother has died illustrates the contrasting content of the documentation and might indicate a lack of preparedness to see the reality of the child.

**Table 4:** Example from the content area Actions by professionals

*“Mother afraid that breastfeeding won’t be enough.”*	
*“Mother thinks that it is fun.”* (Referring to having a baby, author’s comment)	
*“Mother will visit CHS next week.”*	
*“Mother has not contacted CHS.”*	
*“All is well.”*	
*“Mother dead.”*	

### Comparing the two groups

The documentation of the comparison group concerning The Child consisted of about the same proportion of codes (26 %) and included much of the same type of information. Here the child’s own experiences were also neglected. In this group the content area in contrast contained much less data about medical, psychological and behavioural problems as well as parental concerns about the child’s health. Preschool or day care was noted more often (81 %) and leisure was less omitted due to some more, varied leisure activities being noted, e.g, *“swims and practices gymnastics”.* However, in both groups the documentation about the child itself was found to be limited in comparison with most of the other content areas.

In the comparison group the documentation in the content area The Family consisted of 42 %, which made it the dominating area. Contrastingly, the findings in the documentation of this group indicated more stable family situations with fewer separations, new partners or siblings and few legal conflicts. The parental health problems also were fewer and less burdensome as well as parental worries. Mothers were generally highly present in both groups in visits to CHS and in documentation of daily life, while fathers seemed absent in both groups, especially in the case group. Notes on social support in the comparison group were made about existing support, eg, *“grandma and daughter helping mother”* and *“relatives assisting”*. Travelling, seldom noted in the case group, appeared more common in the documentation of this group more, eg, as family holidays.

When it came to Living conditions, a smaller part of data on parental occupation was missing in the comparison group; 29 % of mothers’ and 33 % of fathers’ occupations. The codes in this content area consisted of three percent of the total number of codes and moving, although rarely noted, also took the largest part of the content. Other notes about social structures such as housing, family economy and employment were almost totally absent, but through a few notes such as *“mother working”*, *“mother going to study”, “mother works part time”* and *“father works sometimes”* better living conditions were documented in this group. Still, social structures were found to be essentially invisible also here.

In the comparison group, documentation on the content area Actions by professionals consisted of a smaller part (29 %) but it was also dominated by communication with the family, but the content was quite different as social problems were not at all as prominent here. Furthermore, counselling was more medically oriented although less specialized healthcare was involved. Interestingly, no professional worries at all were documented for the children in this group and only one child was reported to the Social Services. The note “*all is well*” was used 145 times in the documentation of this group, ie, equally frequent as in the documentation of the case group. This comment, or similar, made up most of the physician’s notes in both groups.

## Discussion

**Fig. 3:**
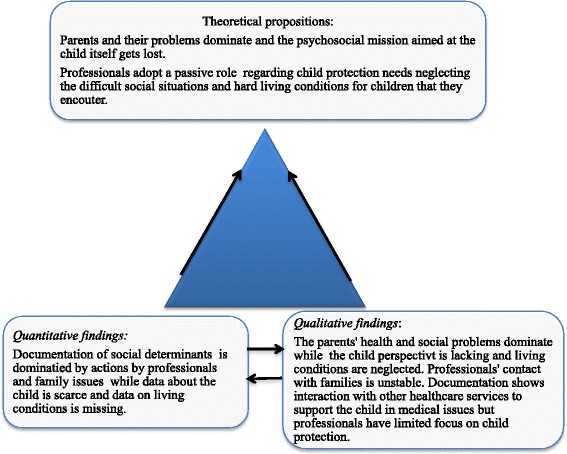
Triangulation of quantitative and qualitative results from the documentation of children in the family foster care group and theoretical propositions

The documentation in the CHS health records of children in family foster care and the comparison group showed similarities concerning documentation of social determinants, but also demonstrated important differences both in space and content. When integrating the quantitative and qualitative analysis using the triangulation metaphor used by Östlund et al. [24], they were found to complement each other. The key findings suggest two theoretical propositions that provide a deeper understanding of the information recorded by professionals in the health records of vulnerable children. Professionals’ documentation is dominated by parental health and social problems as well as interaction with other healthcare and social functions, indicating underlying worries of the professionals and healthcare needs for the children, as well as professionals’ difficulties to make healthcare available for the children. However, the professionals lose the child perspective and adopt a passive role regarding child protection needs in neglecting the difficult social situations that they encounter. Thereby the legal psychosocial mission towards the child itself becomes lost (Fig. 3).

In spite of the broad WHO definition of health, earlier research shows that the health information required by the national standardised CHS and School Health Services health records in Sweden is mainly based on the physical perspectives of health [26]. Similarly, psychosocial information about schoolchildren, has been found missing from health records maintained by Swedish school nurses [27]. Reasons for not documenting difficulties included worries about future misunderstandings, stigmatisation of the child and other ethical concerns and practical barriers, such as record structures focusing on physical health and lack of time [28]. Other studies on CHS and School Health Services health records have shown that free text notes mainly focus on the psychosocial perspective of health, whereas the standardised parts focus on medical health issues [29]. With this in mind, the present study expanded the remit to cover the total content of the CHS health record concerning social determinants of health. The aim of the present study was to develop a better understanding of documentation concerning vulnerable children in, or about to be placed in, family foster care. The results showed that information about living conditions, including parental occupations, was missing to a great extent, confirming earlier research about incomplete data on parents’ occupation in Swedish CHS records [30]. In the study, missing data about the parents’ occupation, especially the fathers, was more common in the health records of children in family foster care than in the comparison group and living conditions was almost totally missing. What are the reasons not paying attention to living conditions in the documentation of vulnerable children, when we know that they deeply affect child health? Is it a matter of omitting sensitive information, is it viewed as being insignificant or outside the professionals’ remit or is it seen as impossible to influence? All these explanations are possible, together with time restraints, and are particularly noticeable when lots of family problems dominate health visits. Studies suggest that parents fail to tell healthcare services about their contact with the Social Services, as they are worried that sensitive information may not be treated as confidential [30]. Some psychosocial information has been shown to be consciously omitted from health records and has taken the form of peer-to-peer verbal communication within and between health and social services [31]. A study of school nurses showed that they had difficulties documenting suspected or verified abuse, even when reports to the Social Services had been made, and some avoided the problem by making temporary notes elsewhere [28]. This might be the case in the present study, but it does not fully explain the low reporting rates to Social Services, unless professionals omitted to document that they had reported to Social Services. However, the finding of the present study is in line with earlier research about CHS professionals not reporting child maltreatment to Social Services [20, 21, 32].

Interestingly, the documentation on social determinants of health in the present study was often more extensive in additional material, such as copies from inpatient clinics and obstetric care. This might be explained by an absolute need for basic information for medical treatment, for example, withdrawal treatment for a newborn baby if the mother is a drug user. In addition, the phrase *“all is well”* was found to be routinely used, more often by physicians. This is interesting, as it possibly reveals professional attitudes towards expected tasks for CHS professionals. Do professionals imagine the visit as a check-up and a somatic inspection and therefore do not consider the child’s situation and living conditions outside the room? Physicians tend to only work a few hours in the CHS and the use of *“all is well”* may further confirm the detachment they feel to their CHS work. This term might be a result of professionals turning a blind eye. It is provocative, but plausible that the professionals use the phrase *“all is well”*, when in reality the vulnerable child’s world should be described as *“all is falling apart”.*

Finally, the deeper analysis of the health records of children in family foster care showed that the child’s own experiences were neglected. For example, leisure activities were seldom mentioned, confirming earlier studies of CHS health records [29]. The lack of this information in the present study might be due to a lack of leisure activities in that group. However, it is also possible that it was a consequence of the social burden in the family and the parents’ problems dominating visits and the documentation. In spite of the extensive documentation about parental problems, which might be expected considering the status of many of the families, it still appears insufficient since the consequences for the child are not being fully acknowledged. Furthermore, information about the living conditions were left out which meant that important factors influencing the life and development possibilities of the child were not paid attention to in the documentation. In the light of the findings, one might ask if another professional approach based on a holistic view of health and knowledge about both the social determinants of health and the Convention on the Rights of the Child, acknowledging how parental problems affect the child’s daily life and development would create more useful engagement that benefits the child.

With the presented results in mind, and using the Global Commission’s recommendation for all countries “to ensure a good start to life for every child,” we suggest education of health professionals about the social determinants of health [3]. Professionals working with vulnerable and maltreated children continuously need to develop their skills and increase their focus on health promotion and preventing harm, as well as ensure that the rights of the child are reinforced [33].

### Methodological considerations

This study employed a mixed methods approach, analysing both quantitative and qualitative data, and thereby capitalising on the benefits of both methods. Using a matched comparison group clarified differences between the CHS documentation for children in family foster care and those not. Investigating the CHS professionals’ documentation provided valuable information, as it exposed the routines and possible attitudes of a professional group that has both the opportunity and the obligation to make a difference when children are in vulnerable positions. It might be considered a limitation that only one of the authors coded the data. However, the coding process was continuously discussed and negotiated between the first and the last author. A number of study subjects were lost as an up-to-date could not be found and some parents refused to consent. In addition, some health records could not be found, probably because of the children moving frequently and a deficient archiving system. The fairly high number of records lost due to lack of consent implies a potential selection bias. These records probably represented children with worse living conditions, with more parental problems and frequent moves. If they had been included this would probably have resulted in even bigger differences between the two groups. The missing health records also demonstrated the known problems in carrying out studies of vulnerable groups of children. Furthermore, this study included a relatively small sample of health records from a specific geographical area limiting the generalizability of the study findings. In addition, health records do not tell us everything about what professionals know and how they perform, a point that has been demonstrated by other researchers [31]. Even though the data was collected in 2008, there is no indication that the documentation in the CHS has changed in a comprehensive way since then.

## Conclusions

By employing a mixed methods research approach, this study presents both broad and unique data on what Child Health Services professionals document concerning social determinants of health in the health records of children in general and specifically for children in family foster care. Parents and their problems dominate and the psychosocial mission aiming for the best of the child gets lost. Professionals adopt a passive role regarding child protection needs neglecting the difficult social situations and living conditions for children that they encounter.

The study identified that the documentation only partly showed professionals’ actions and also highlighted infrequent reporting by professionals to Social Services about children suspected or verified to be maltreated or neglected. The findings underline the fact that professionals need to interact more with Social Services and might benefit from understanding more about social determinants of health and how to integrate them into the professional provision of Child Health Services. Training and education about social determinants of health might sharpen professionals’ competence and ability to identify children at high risk of maltreatment or neglect. Documentation, at its best, mirrors the child’s situation and creates the opportunity to develop a broad understanding of the physical, mental and social health of each child. It can pave the way for action concerning health and social issues that matters. The results also highlight the need to develop professionals’ understanding of their important role and to have a more profound focus on children’s rights.

## Abbreviations

CHS, Child Health Services; CRC, Convention on the Rights of the Child
